# Mercury in aquatic ecosystems of two indigenous communities in the Piedmont Ecuadorian Amazon: evidence from fish, water, and sediments

**DOI:** 10.1007/s10646-024-02764-w

**Published:** 2024-06-07

**Authors:** Daniel Escobar-Camacho, Daniela Rosero-López, Melany Ruiz-Urigüen, Karla S. Barragán, Natalia Carpintero-Salvador, José R. Daza, Allison Aldous, Silvia Benítez, Timothy Tear, Andrea C. Encalada

**Affiliations:** 1 Laboratorio de Ecología Acuática, Instituto BIOSFERA, Universidad San Francisco de Quito USFQ, Quito, 170150 Ecuador; 2 Core Lab de Ciencias Ambientales, Universidad San Francisco de Quito USFQ, Quito, 170901 Ecuador; 3 Ingeniería Ambiental, Colegio de Ciencias e Ingenierías, Universidad San Francisco de Quito USFQ, Quito, Ecuador; 4 The Nature Conservancy, Calgary, AB Canada; 5 The Nature Conservancy, Quito, Ecuador; 6 Biodiversity Research Institute, Portland, ME USA

**Keywords:** Total mercury (THg), Fish, Fluvial sediment, Water, Ecuadorian Amazon

## Abstract

Mercury is a highly toxic element present in water, soil, air, and biota. Anthropogenic activities, such as burning fossil fuels, mining, and deforestation, contribute to the presence and mobilization of mercury between environmental compartments. Although current research on mercury pathways has advanced our understanding of the risks associated with human exposure, limited information exists for remote areas with high diversity of fauna, flora, and indigenous communities. This study aims to deepen our understanding of the presence of total mercury in water, sediments, and fish, within aquatic ecosystems of two indigenous territories: Gomataon (Waorani Nationality) and Sinangoé (Ai´Cofán Nationality) in the Ecuadorian Amazon. Our findings indicate that, for most fish (91.5%), sediment (100%) and water (95.3%) samples, mercury levels fall under international limits. For fish, no significant differences in mercury levels were detected between the two communities. However, eight species exceeded recommended global limits, and one surpassed the threshold according to Ecuadorian legislation. Piscivore and omnivore fish exhibited the highest concentrations of total mercury among trophic guilds. Only one water sample from each community’s territory exceeded these limits. Total mercury in sediments exhibited greater concentrations in Gomataon than Sinangoé. Greater levels of mercury in sediments were associated with the occurrence of total organic carbon. Considering that members of the communities consume the analyzed fish, an interdisciplinary approach, including isotopic analysis, methylmercury sampling in humans, and mercury monitoring over time, is imperative for a detailed risk assessment of mercury exposure in Amazonian communities.

## Introduction

Mercury (Hg) contamination is a prominent and growing global concern (United Nations 2013), and human exposure to Hg is currently considered a significant public health issue (Park and Zheng. 2012). All forms of mercury are toxic; however, organic mercury, particularly in the form of methylmercury (MeHg), is known for being one of the most harmful elements to humans. MeHg is known for its propensity to bioaccumulate and increase concentration through the trophic chain, posing a risk to top-level consumers, including humans, where it affects mainly the central nervous system. Acute intoxication with MeHg can lead to symptoms such as loss of coordination, motor disturbances, or progressive deterioration of visual and tactile senses (WHO 2019; Crespo-López et al. 2021).

MeHg in reducing environments is produced by transforming oxidized mercury (Hg2+) to MeHg by methanogenic aquatic bacteria found in periphyton and substrates in reducing environments (Achá et al. 2011). Unlike the inorganic forms of mercury, MeHg is retained better by organisms in the food web (Morel et al. 1998), biomagnifies, and reaches higher concentration in upper-trophic-level fish. Although studying mercury in its organic form (MeHg) is highly relevant due to its toxicity and the negative impacts it represents on people and biota, the detection of all inorganic forms of Hg (which could later be transformed into MeHg), such as those reported for soils composition (Lima et al. 2022) and atmospheric deposition through the forest canopy (Gerson et al. 2022), is critical to the understanding of the contribution of human activities to the presence of inorganic mercury in the environment (Lasorsa and Allen-Gil. 1995). Furthermore, mercury concentration strongly correlates to nutrient input, such as all forms of organic carbon (i.e., TOC, DOC), resulting from decomposition (Erickson and Lin. 2015; Bravo et al. 2018).

In the Amazon River Basin, mercury has been found in the organic (MeHg) and inorganic forms (Hg^0^, Hg^2+^) in sediments of different aquatic habitats (Crespo-López et al. 2021). Recent evidence suggests that inorganic and organic mercury will likely increase in the Amazon tropical rainforest due to anthropogenic activities like artisanal mining, extensive biomass, and deforestation (Gerson et al. 2022). Artisanal-small scale gold mining (ASGM) has been recognized as an important contributor to the presence of mercury in the Amazon (Gerson et al. 2022). Concomitantly, the climate and hydrology in the headwaters of the Amazon River Basin, which are distinctively dynamic, contribute directly to weathering and become a significant natural mechanism for elements like mercury to enter the trophic network (Siqueira et al. 2018) and to reach humans through fish consumption (Roulet and Lucotte 2001).

In addition to anthropogenic activities, Amazonian soils have also been identified as a source of natural mercury, even in areas without human perturbation (Siqueira et al. 2018; Lima et al. 2022). Mercury mobilization from terrestrial to aquatic ecosystems and then to biota can increase with the duration and frequency of hydrological events through the wetting of habitats (i.e., igapó and várzea) (Mainville et al. 2006). Notably, mercury in natural systems have been linked with total organic carbon (TOC) and dissolved organic carbon (DOC), underscoring the importance of organic matter in the dynamics of liberating, and retaining mercury forms in soil sediments. Therefore, mercury in the Amazon results from natural origins, mobilization, and anthropogenic activities (Pouilly et al. 2012; Gerson et al. 2022). Despite these sources, human exposure to mercury in some Amazonian indigenous communities is strongly associated with fish consumption (Carvalho et al. 2019; Gonzalez et al. 2019).

The Ecuadorian Amazon is home to 11 indigenous nationalities living in one of the most biodiverse regions worldwide (CONFENIAE 2023). The Ecuadorian Amazon harbors has more than 800 freshwater fish species distributed in three main river basins: Napo, Pastaza, and Santiago Rivers (Barriga 2012). This diversity might be underestimated because research suggests ~744 species from the Napo River alone (Jézéquel et al. 2020). Like in other Amazonian countries, artisanal fisheries are of utmost importance for Ecuadorian Indigenous communities settled on riverbanks and nearby flooded forested areas, where fish is the primary source of protein and fat in their diet (Berlin and Markell 1977; Dufour 1983; Sirén 2004, 2011). At the same time, some regions in the Northern Ecuadorian Amazon have experienced a long history of oil exploitation activities, particularly in lacustrine systems (i.e., Cuyabeno Natural Reserve) and mainstem rivers (i.e., Aguarico, Coca, Payamino, Dué, Napo) (Sierra 2000; Encalada et al. 2019b; Lessmann et al. 2019), where research has evidenced heavy metal pollution (i.e., mercury) threatens aquatic ecosystems and human communities (Webb et al. 2004; Mainville et al. 2006; Slik et al. 2015). In the last decade, artisanal and small-scale gold mining (ASGM), as well as sand/gravel mining have been added to the list of threats to aquatic ecosystems in the Ecuadorian Amazon (Mestanza-Ramón et al. 2022). ASGM activities established in mainstem rivers like the Aguarico, Punino, and Napo River disturb riparian vegetation and riverine habitats (Encalada et al. 2019b).

To assess the risk of mercury exposure to Indigenous communities in the Ecuadorian Amazon, we analyzed total mercury (THg) content in water, sediment, and fish samples from two communities located in the transition zone between the Amazonian lowlands and piedmont forests of the Napo River Basin. One community, Gomataon, belonging to the Waorani Nationality, lacks any mining records in its vicinity. In contrast, Sinangoé, belonging to the Ai´Cofán Nationality, is surrounded by sand/gravel mining and artisanal, small and medium-scale gold mining, resulting in more visible signs of disturbance. In this research, we hypothesized that greater THg concentrations in aquatic ecosystems’ compartments (i.e., fish, sediments, water) would be found in communities with mining activities. Additionally, because mercury methylation rates are higher where there is more organic carbon and a reducing environment, we secondarily hypothesized that mercury concentrations would be higher where there was higher TOC in sediments, regardless of the presence of mining. To test our hypotheses, we characterized the presence of THg and variations in water, sediments, and fish in both indigenous communities. In addition, to further assess risk exposure, we examined THg concentrations and recommended thresholds for fish consumption and sediments. Results from this study will allow us to assess THg presence and concentrations that meet local and international health standards, providing insight into the risk for indigenous communities in the Ecuadorian Amazon. Mercury concentrations in aquatic ecosystems have critical consequences for food security, community health, and the conservation of remote indigenous territories.

## Materials and methods

### Study area

**Fig. 1:**
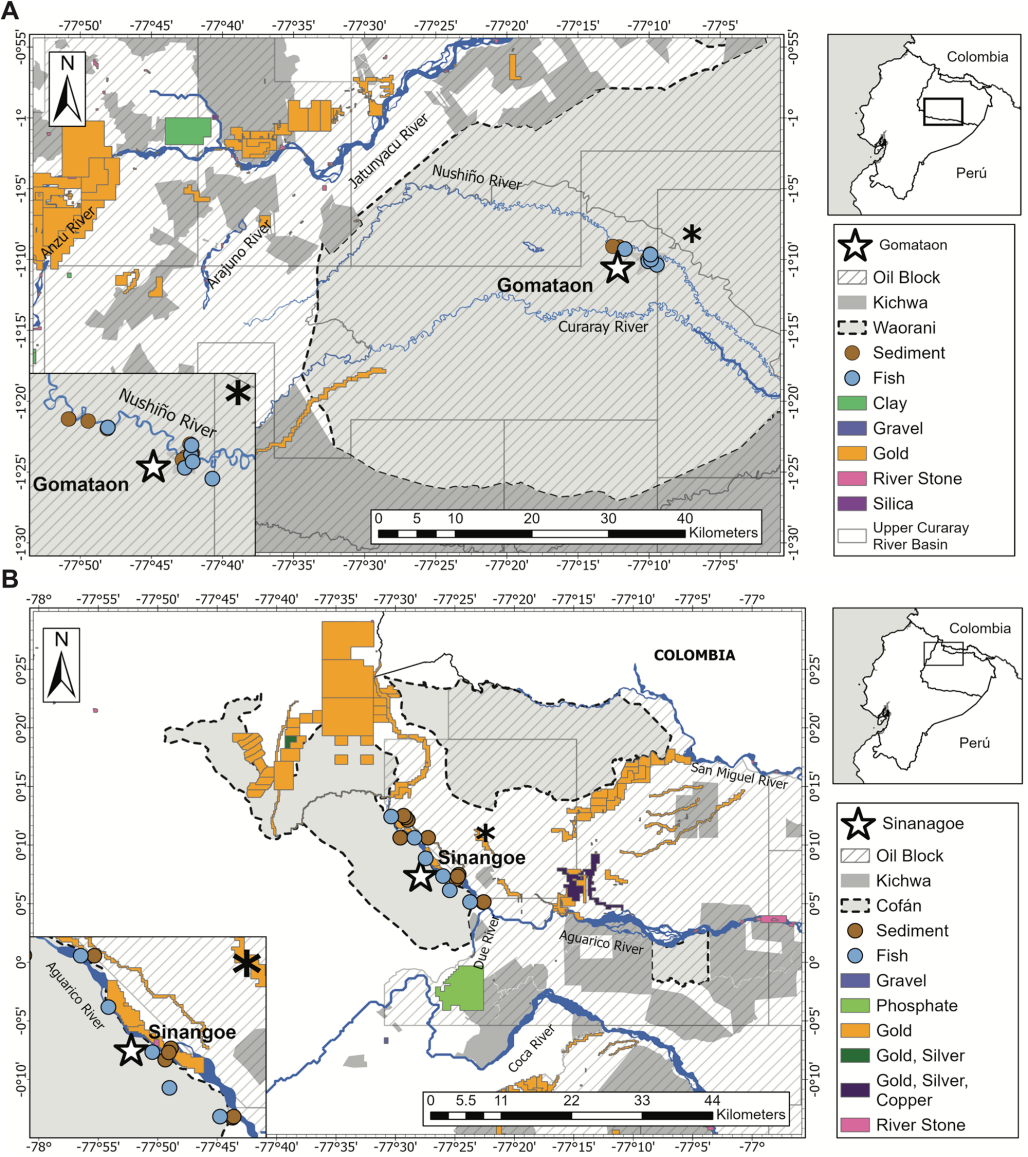
Maps of the Northeastern Ecuadorian Amazon study area. **A** Depicts Gomataon’s location at the Upper Curaray River Basin limit. **B** Depicts Sinange’s community limits in the Aguarico River Basin. Indigenous community territories of Waorani and Cofán nationalities are shown in gray and dashed lines, while other indigenous nationalities’ territories are shown in dark gray. Superimposed oil blocks are depicted by squared polygons and diagonal lines, and mining concessions by different colors for each type. Blue circles indicate sampling sites for fish, and brown circles indicate sampled sites for sediments and water

For this research, we worked with indigenous communities located in the Nushiño and Aguarico rivers, both tributaries of the Napo River in the Amazon Basin (Fig. 1). The Nushiño River, in the south region of the Napo River basin in Ecuador, connects Waorani and Kichwa territories through the fluvial scape where no road infrastructure has been developed yet (Fig. 1A). The Aguarico River presents several human settlements, extensive land use change for agriculture, fossil fuel activities, ore-mining, and hydropower (Fig. 1B) (Lessmann et al. 2019; Mestanza-Ramón et al. 2022). The indigenous communities in this study have managed their territories to conserve forest and water. However, the pressure of increasing mining activities has drawn particular attention to the level of risk indigenous communities are exposed in gold mining and the use and presence of mercury. Throughout the manuscript, the communities of Gomataon and Sinangoé are differentiated using mercury in artisanal mining; where Gomataon is a no-mining zone, and Sinangoé is an artisanal mining zone. Sinangoé is also threatened by large-scale legal mining in the surroundings of the Aguarico River.

Gomataon: This Waorani community, at 225 m a.s.l., is settled on the left riverbank of the Nushiño River, a meandering white-water river with a 2 to 8 m depth and 10 to 50 m width. The Nushiño River is the northern tributary of the Curaray River, which drains into the Napo River in Perú, and then joins the Amazon River. The headwaters of the Nushiño River remain primarily intact, with a few human settlements on its banks (Fig. 1A). No infrastructure has been built along the Nushiño River mainstem and Gomataon does not engage in alluvial mining activities within its territory. Most of the riparian vegetation remains conserved in the community, which is surrounded by primary and secondary evergreen rainforest and flooded palm forest from the alluvial Amazon floodplain. Aquatic ecosystems include rivers, streams, waterfalls, swamps, and oxbow lakes. The territory is located within the Yasuní Biosphere Reserve. The Nushiño fluvial system is a free-flowing river of high importance for the conservation of freshwaters and aquatic biodiversity (Chuctaya et al. 2021,2023). It has been part of a local initiative to protect fluvial systems and to support the creation of freshwater- protected areas (Koning et al. 2020; Higgins et al. 2021). A process of community decision-making led to the creation of the first *Natural Fluvial Reserve* in Ecuador (other effective area-based conservation measures, OEMC) intending to protect aquatic ecosystems and biodiversity of the Nushiño and Curaray Rivers (Encalada et al. (in prep.). The Gomataon community, like most indigenous territories and protected areas in Ecuador, is settled over oil concession blocks that have been established in the whole Ecuadorian Amazon (Block N° 22) (Fig. 1A). Within these blocks, the government owns exploration wells that can be bid by oil companies to obtain exploitation rights. In 2021, rock mining started in the headwaters of rivers adjacent to the Nushiño River: Arajuno, Anzu, and Jatunyaco (upper Napo River basin) (ARCOM 2020).

In addition, in 2021, an international demand for balsa wood increased the exploitation of secondary forests within the Waorani territory. The community consists of 28 people; 15 are permanent residents in the territory, and the remaining members move between their territory and the main cities in search of education, health care, and job opportunities. Access to the community of Gomataon and 36 other indigenous communities is possible only by boat through the Nushiño and Curaray rivers. A hose system provides drinking water with limited filtration and chlorination at the source point. The community does not consume water from the Nushiño River. There is no sanitation, and the primary way of disposal are latrines. The community’s livelihood is based on local sporadic scientific expeditions and logging. The community diet, as well as for most Waorani people, includes yuca cassava, plantain, palm, and peanut (i.e., traditional crops), and for protein, they consume fish, birds, and mammals. Fishing is one of the cultural activities the community still practices using traditional techniques.

*Sinangoé:* This Ai´Cofán community, at 525 m a.s.l., is settled in the riverbanks of the Aguarico River, a large meandering white-water tributary of the Napo River (Fig. 1B), with 0.5–4 m of depth and 20–40 m of width in this area. The territory of the Sinangoé community is dominated by evergreen piedmont forest and includes aquatic ecosystems such as rivers, waterfalls, and streams. No infrastructure has been built in the Aguarico River. The community livelihood is based on local tourism, aquaculture ponds for fish (“cachamas” *Piaractus brachypomus* and “paiche” *Arapaima gigas*), agriculture for local consumption, hunting, and artisanal mining. Around the Sinangoé settlement, most native riparian vegetation has been removed. The Sinangoé population comprises of 50 families, of whom ~195 individuals are permanent residents and ~60 people move between the closest city and the community. Access to the community is restricted to boats that cross the Aguarico River. Electricity through the national grid provides energy and internet access to several households and the elementary school. Water is supplied through a hose system from springs at ~3.5 km from the community. The community does not engage in alluvial mining activities. Sinangoé does not consume water from the Aguarico river, drinking water is filtered and chlorinated before reaching the school, the community dining, and a few households. No proper sanitation exists in the community, and latrines are the only way of disposing of human -waste disposal.

The territory is in close proximity to the oil concession limits of Block N^o^ 11. Up until 2020, the Ecuadorian government authorized 138 mining concessions covering an area of at least 393.82 km^2^ surrounding Sinangoé (estimated from Fig. 1B). These concessions encroach upon tributary rivers and the Aguarico River itself within the Sinangoé territory (Fig. 1B). In addition, some members of Sinangoé perform artisanal gold mining in their territory. This ASGM activity uses metallic mercury to extract gold by amalgamation (Pers. com.) and uses dredges and water pumps placed in the riverbanks to wash and separate the gravel-sand-ore mix. Once the ore is separated by decantation, it is collected in a washing pan and combined with the metallic mercury. Finally, through boiling mercury, it evaporates and leaves the gold nuggets. The proportion of the Sinangoé population involved in ASGM and the frequency of these activities is unknown.

### Sampling fish, water, and sediments

Sampling was designed through a community-engaged and participatory approach. Sampling sites were selected according to community members’ criteria of preference sites for fishing. We prioritized localities based on fishing frequency, ancestral knowledge, and accessibility. Gomataon: swamp, oxbow lake, streams, and the main river, and Sinangoé: streams and rivers (Table 1, Fig. 1). The diversity of water bodies itself introduced variation, however, we wanted to capture all potential sites of mercury presence that are related to fishing activities. We measured in situ pH, conductivity, temperature, dissolved oxygen (DO) concentration, and saturation using a YSI PRO DSS® multiparameter, previously calibrated.

**Table 1:** Environmental conditions and location details of aquatic ecosystems sampled for mercury (THg) presence and concentration in two indigenous communities of the Ecuadorian Amazon

Habitat	Locality	Lon. (W)	Lat. (N)	Alt. (m)	pH	Cond. (uS/cm)	Temp. (^o^C)	DO (mg/l)	DO (%)
Gomataon									
Main river	Nushiño River up	−77.16766	−1.16501	272	6.13	32.7	24.4	6.67	79.9
Main river	Nushiño River down	−77.195	−1.15474	278	6.24	31.8	24.1	6.71	81.1
Stream	Gomataon stream	−77.16738	−1.16886	293	6.79	28.8	23.6	7.48	88.3
Stream	Mancaompare stream	−77.15751	−1.17273	289	6.07	29.3	23.7	7.4	87.5
Oxbow lake	Oxbow lake	−77.16682	−1.16395	269	6.58	34.9	24.7	6.57	79.2
Swamp	Swamp	−77.16493	−1.16071	281	6.34	34.8	28.1	3.68	48.1
Sinangoé									
Main river	Aguarico River	−77.43153	0.12521	510	7.26	92.52	23.3	7.23	83.2
Tributary river 1	Candué river 1	−77.39604	0.08562	460	6.99	32.82	23.5	6.41	78.5
Tributary river 2	Candué river 2	−77.39544	0.08593	460	7.04	80.59	24.2	6.15	75.5
Stream	Sebastiana stream	−77.50601	0.20731	578	7.21	131.70	24.5	7.14	81.4

We conducted the field campaigns between April and June of 2022 (at the end of the wet season). We sampled water and sediments using established protocols (see below). We collected fish using the local community’s typical techniques, including hook lines, gill nets, cast nets, and hand nets. Most techniques targeted medium to large fish. We collected all fish, however, we focused on species usually consumed by the community. Each aquatic ecosystem was sampled once for 4 h, except for the oxbow lake in Gomataon, where the gill nets were retrieved after 12 h. Once we collected fish, we photographed most individuals and visually identified specimens to the lowest possible taxonomic level. Specimens were coded, measured, and weighted before cutting 10–40 g of epaxial muscle above the lateral line between the head and the origin/end of the dorsal fin. For all fish manipulation we used nitrile gloves and ceramic knives to avoid cross-contamination with other metals. For Loricariids, given their rigid bony plates, we extracted the muscle from the area between half of the dorsal fin and the caudal peduncle. All muscle samples were weighted, labeled, stored in Whirl-pack® plastic bags, and frozen until processed in the laboratory.

We collected 250 g of sediments in four replicates, in each aquatic ecosystem listed above. Due to high velocities and depths in mainstem rivers, we sampled only the margin where the community is settled. We sampled upstream and downstream of the community. We sampled in the middle part of the stream and the left and right margins in wadable streams. In the oxbow lake and the swamp, we sampled along the shore in three sites covered with water throughout the year. We did not sample in the middle of these water bodies because they are not accessible by boat. All samples were taken with small plastic shovels, placed in plastic bags (Whirl-Pak®) labeled, and then preserved frozen until laboratory analysis.

For the assessment of mercury presence and concentration in water, a volume of 60 ml was collected and filtered through a 0.45 µm filter attached to a disposable syringe into polyethylene bottles. Next, 0.3 ml of 0.1% nitric acid (HNO_3_) was added to the filtered water samples. The labeled samples were then preserved in dry ice to transport to the laboratory. Upon arrival, samples were maintained at −20 °C until ready for processing and analysis. To avoid cross-contamination, all samples, fish, sediment, and water were carefully handled using nitrile gloves. Samples were analyzed in the Core Lab of Environmental Sciences at Universidad San Francisco de Quito.

### Laboratory analyses

#### Total mercury analysis

For fish, samples were freeze-dried over a period of three to 4 days utilizing Labconco lyophilizer (model FreeZone 2.5 L) at −50 °C and 0.1 mBar of pressure. Water content was calculated for each sample. Dried samples were then ground, sieved using an N° 35 mesh and weighted 40 mg on a nickel sample boat. Triplicate measurements for total Hg were carried out following the EPA method 7473 by gold-amalgamation thermal decomposition using a Milestone dual cell direct mercury analyzer, DMA–80^®^. For quality control, we used certified reference material (CRM) NRC-CNRC DORM-04 with a certified value of 0.412 ± 0.036 mg/kg, and it showed an average recovery of 97,17%. The CRM was measured every 20 runs with a relative standard deviation (RSD) of 3%. The method detection limit for fish was 0.48 µg/kg, determined by measuring 5 sample boats blanks

Sediments samples were freeze-dried, sieved, and analyzed in triplicate for total Hg, as explained above, following the EPA method 7473 in a DMA–80^®^.40 mg of sample were used for each measurement. For quality control, we used CRM NIST 2709 San Joaquin soil, with a recovery of 102%. The method detection limit for sediments was 0.48 µg/kg, determined by measuring calcinated sediment as blanks

Previously filtered and acidified water samples, were processed by weighing 100 µL of sample on a quartz boat and a silica substrate, then analyzed in triplicate for total Hg following the EPA method 7473 in a DMA–80^®^. For quality control, blanks were run, and 5 samples were spiked with 50 µg/L mercury using standard Inorganic Ventures AAHg10 of 10 µg/mL in order to quantify a recovery percentage; and it had an average recovery of 92.67% and an RSD of 3.34%. The detection limit for water was 0.5 µg/L, we used deionized water as blanks

#### Total organic carbon analysis

The determination of Total Organic Carbon (TOC) was determined in sediments following the EPA 9060 method, using a Shimadzu SSM 5000A analyzer. A quality control standard consisting of 3% of dextrose (n = 4) was used, resulting in a recovery of 85.82% and an RSD of 7,62%. The detection limit of TOC in sediments was 0,008%. %

In water samples, total dissolved organic carbon was determined using an ASI- L and a TOC-L Shimadzu analyzer. For quality control, the standard used was the ERA 978 QR of 50 mg/L (n = 6) with a recovery of 102,35%. The detection limit of TOC in water was 0,6 mg/L.

### Data analysis

To understand THg variation across fish and mercury predictors such as trophic guilds and size (Standard Length: SL), we assigned the trophic category to each fish using FishBase.org (Froese and Pauly 2023) and relevant literature of each specific species (Tables S1–2). Whenever this information was unavailable, or data were missing from the database, we assigned the trait/value based on closely related species.

First, because different fish species were caught in each community, we conducted an Analysis of Covariance (ANCOVA). The data from fish tissue were log-transformed to meet assumptions of linear regression. For each community, linear models were constructed with THg as the response variable, trophic guilds as the explanatory variable, and type of water body and fish size (SL) as covariates. For Gomataon, the model used as category reference detritivores and Oxbow Lake for trophic guilds and ecosystems. For Sinangoé, the model used as reference detritivores and Tributary Rivers for trophic guilds and ecosystems, respectively.

Second, to test if the presence/absence of mining in the communities would lead to differential risk exposure to mercury, we used mixed-effect models on THg concentration in fish tissues and their respective components. This was done using function “lmer” of the “lme4” package (Bates et al. 2015). The model included ecosystem type, trophic guild, and SL as random effects and the presence or absence of mining as a fixed effect. To assess the significance of the effect of the presence/absence of mining, the model was compared with an ANOVA to a null model with a random intercept. This analysis was conducted for the entire dataset. All models were checked for homoscedasticity and normality of residuals.

To test for differences between sediment samples of different ecosystems in each community, we employed a non-parametric Kruskal-Wallis-test followed by a Holm-Bonferroni corrected Wilcoxon pairwise comparison. Later, we tested for differences in THg between sediment samples from both communities. For this, we performed a robust linear regression for non-parametric data to test for differences in THg between sediment samples from both communities. We used the “rlm” function from the “MASS” package (Venables and Ripley 2002), where THg was the response variable, the communities were the explanatory variable, and ecosystem type was the covariable. Finally, to understand the potential relationship of carbon concentration with THg in sediments, we analyzed TOC from sediment samples from different aquatic habitats and its association with THg concentrations through an exponential regression. All statistical analyses were perfomed using R software (R Core Team (2014)).

To better understand mercury exposure of the indigenous communities, we compared THg concentration from fish to local and international regulation thresholds. These thresholds differ according to fish intake and/or mercury forms. The Ecuadorian legislation establishes a MeHg maximum level in fish of 500 µg/kg for non-predatory fish species and a concentration of 1000 µg/kg for predatory fish (NTE INEN-codex 193:^2013^). We also compared THg concentrations to the World Health Organization (WHO 2019) and the US Environmental Protection Agency (EPA) (USEPA 2001). The WHO sets the limit for fish people consume to 500 µg/kg based on a weekly 200 mg intake (AMAP/UN Environment 2019) and the USEPA 300 µg/kg (Buck et al. 2019). For sediment, the mercury threshold is 486 µg/kg, as suggested by The Canadian Guidelines for the Protection of Aquatic Life (CCME 1999). For water, the Ecuadorian legislation allows a limit of 6 µg/L for human consumption (TULSMA 2015), and the USEPA has a maximum permissible level of 2 µg/L for drinking water (USEPA 2022).

### Local community surveys

For this research, we co-designed the surveys and the research with local community members after critical discussions about mercury pollution in local riverscapes and fish. Many members participated in the meetings and during the sampling and analyses. The survey was performed to familiarize the researchers with the relationship between fish and indigenous communities. We surveyed (File S1) community members who participated in previous discussions about the project and consented to it. These surveys were intended to learn more about their fishing and fish consumption habits, but not to quantify fish consumption. We surveyed eight individuals from Gomataon and 14 from Sinangoé; i.e., 40% and 7% of their population respectively. Surveys asked about how frequently they ate fish and how they perceived anthropogenic perturbances in their territory.

## Results

### Fish sampling

A total of 202 individual fish belonging to 40 species from different river basins were analyzed.

In Gomataon, in the Nushiño River, we captured fish species comprised four orders: Siluriformes, Characiformes, Cichliformes, and Myliobatiformes distributed in 12 families and 27 species (Figs. S1–2, Table S1). The most abundant families were Pimelodidae, Loricariidae, and Characidae, and the most numerous species were *Pimelodus blochii*, *Aphanotorulus unicolor*, and *Aequidens tetramerus*. In Sinangoé, in the Aguarico River, captured fish comprised three orders: Siluriformes, Characiformes, and Cichliformes, distributed in 8 families and 14 species. The most abundant families were Characidae and Prochilodontidae, and the most numerous species sampled were *Astyanax* sp, *Prochilodus nigricans*, and *Salminus hilarii* (Figs. S1–2, Table S2). At least three trophic guilds were caught in six out of seven aquatic ecosystems (Table S3).

### Mercury in fish

**Fig. 2:**
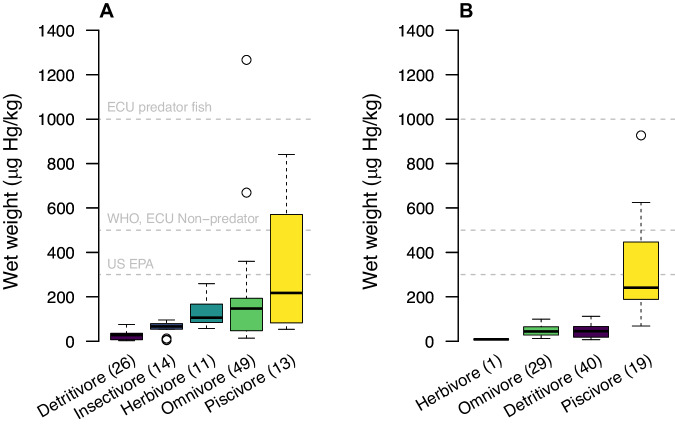
Comparison of THg concentrations in fish tissue across different foraging guilds from (**A**) Gomataon and (**B**) Sinanogé. Open circles are outliers, while horizontal lines represent the first, second (median), and third quartiles. Background gray horizontal lines indicate THg permissible limits in fish established by: Ecuadorian Legislation (for predatory fish), World Health Organization (WHO) and Ecuadorian Legislation (for non-predatory fish), and the United States Environmental Protection Agency (US-EPA). Two outliers from (**A**) of 1596.45 and 2428.17 µg Hg/kg are not shown

**Fig. 3:**
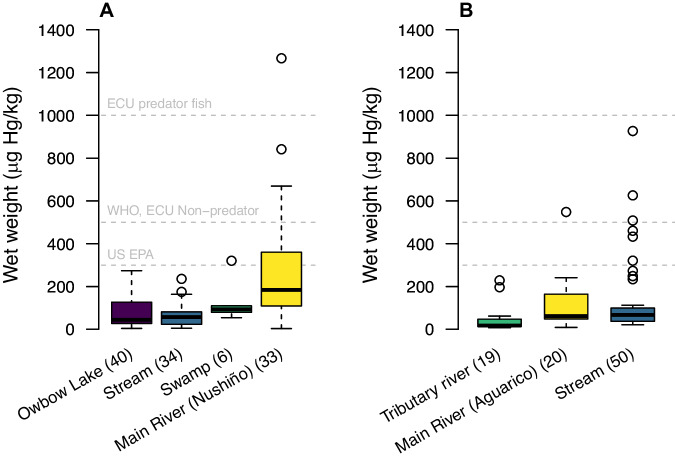
Comparison of THg concentrations found in fish tissue across different aquatic ecosystem samples in Gomataon (**A**) and Sinangoé (**B**). Open circles are outliers, while horizontal lines represent the first, second (median), and third quartiles. Background gray horizontal lines indicate THg permissible limits in fish established by: Ecuadorian Legislation (for predatory fish), World Health Organization (WHO) and Ecuadorian Legislation (for non-predatory fish), and the United States Environmental Protection Agency (US-EPA). Two outliers from (**A**) of 1596.45 and 2428.17 µg Hg/kg are not shown

**Fig. 4:**
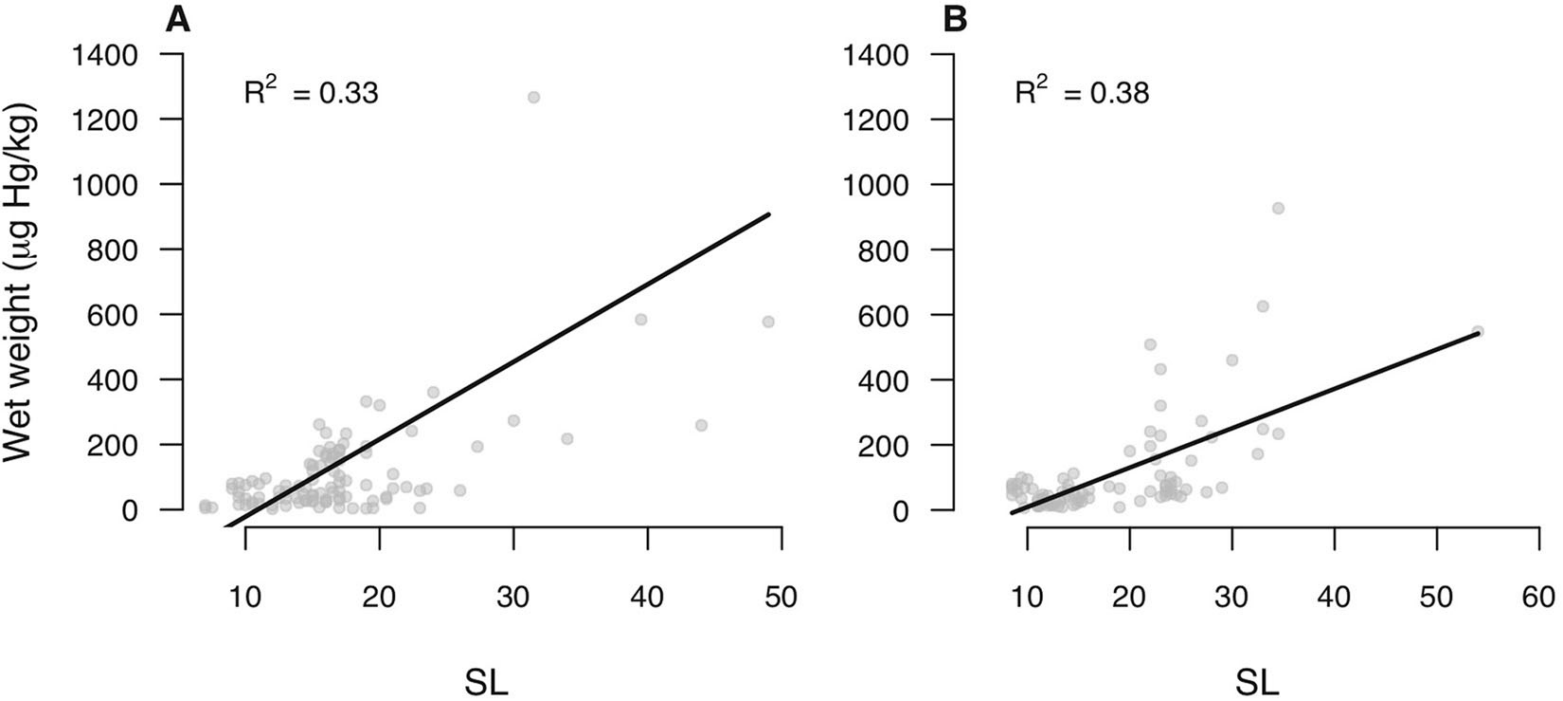
Fish Wet weight and Standard Length (SL) relations for THg concentrations, obtained through Rank-based estimation model (Kloke and McKean 2012), present in fish captured in Gomataon (**A**) and Sinangoé (**B**). Two outliers from (**A**) of 1596.45 and 2428.17 µg Hg/kg are not shown

Total mercury was detected in all fish samples from both communities (Tables S4–5). Trophic guilds were categorized in five categories and exhibited concentrations between 2.78 and 2428.17 Hg (µg/kg) (Table 2). Piscivores exhibited the highest THg concentrations in both communities, while detritivores and herbivores showed the lowest in Gomataon and Sinangoé, respectively. For Gomataon, the ANCOVA model was significant (F-statistic = 20.02, p < 0.001***) and explained 63.02% of the variance in THg concentrations. Trophic guilds demonstrated significant effects (Omnivores: Estimate = 1.719, p < 0.001***; Insectivores: Estimate = 1.727, p < 0.001***; and Herbivores: Estimate = 1.113, p = 0.014*), with omnivores exhibiting the highest impact, suggesting greater concentrations (Table 2) (Fig. 2A). Piscivores did not show a statistically significant effect (Estimate = 1.71, p = 0.088). Aquatic ecosystems did not show significant associations with THg concentrations (Main River: Estimate = 0.402, p = 0.072; Stream: Estimate = −0.379, p = 0.179; Stream: Estimate = 0.722, p = 0.259) (Fig. 3A). Fish size showed a positive relationship with mercury concentration (SL: Estimate = 0.083, p < 0.001***) (Fig. 4A).

**Table 2:** Results from THg were found in the wet weight of fish tissues

Foraging Guild	n	Hg (µg/kg)			
		Mean	SD	Max	Min
Gomataon					
Detritivore	26	26.54	18.52	75.11	2.78
Herbivore	11	130.57	68.34	258.79	57.64
Insectivore	14	59.09	30.64	95.94	4.89
Omnivore	49	239.08	427.74	2428.17	13.95
Piscivore	13	295.55	261.96	841.09	54.00
Sinangoe					
Detritivore	40	47.52	29.63	112.19	7.06
Herbivore	1	8.48	–	–	–
Omnivore	29	48.11	23.25	99.23	12.10
Piscivore	19	326.01	210.09	926.72	68.66

For Sinangoé, the model was also significant (F-statistic = 49.83, p < 0.001***) and explained 76.9% of the variance in THg concentrations. The results revealed significant effects for several covariates. Trophic guilds demonstrated significant effects (Herbivores: Estimate = −1.488, p = 0.004**; Piscivores: Estimate = 01.447, p < 0.001***) with herbivores exhibiting a significant adverse negative effect, suggesting lower THg concentrations. Omnivores did not show a statistically significant effect (Estimate = 0.149, p = 0.279) (Fig. 2B). Aquatic ecosystems also played a role with fish collected from the Main River (Aguarico) having a positive effect (Estimate = 0.479, p = 0.006**) as did fish from stream habitats (Estimate = 0.762, p < 0.001***) (Fig. 3B). Fish size showed a positive relationship with mercury concentration (SL: Estimate=0.040, p < 0.001***) (Fig. 4B).

Our mixed-effect model results indicate that, based on the current data, mining activities does not pose a risk to mercury exposure in fish when considering different trophic guilds, type of aquatic ecosystems and fish sizes. Specifically, the model comparing mining presence/absence to the null model did not show a significant difference (Chisq = 1.1797, Df = 1, p = 0.277), suggesting that mining presence/absence does not contribute to explaining variation in mercury levels among fish. The mean mercury concentration in fish from mining absence (Gomataon) was 163.81 µg Hg/kg, compared to 106.73 µg Hg/kg in mining presence (Sinangoé).

Of all fish tissue analyzed, only 8.5% of samples equivalent to 8 species surpassed thresholds of the US EPA (300 µg Hg/kg), and 5.5% of samples equivalent to 5 species had THg concentrations above the WHO (500 µg Hg/kg) limit (Fig. S3). Only one species (1.5% of analyzed tissues), the omnivore *Calophysus macropterus*, presented THg concentrations above the Ecuadorian legislation (1000 MeHg µg/kg for predatory fish).

### Mercury in water and sediments

**Fig. 5:**
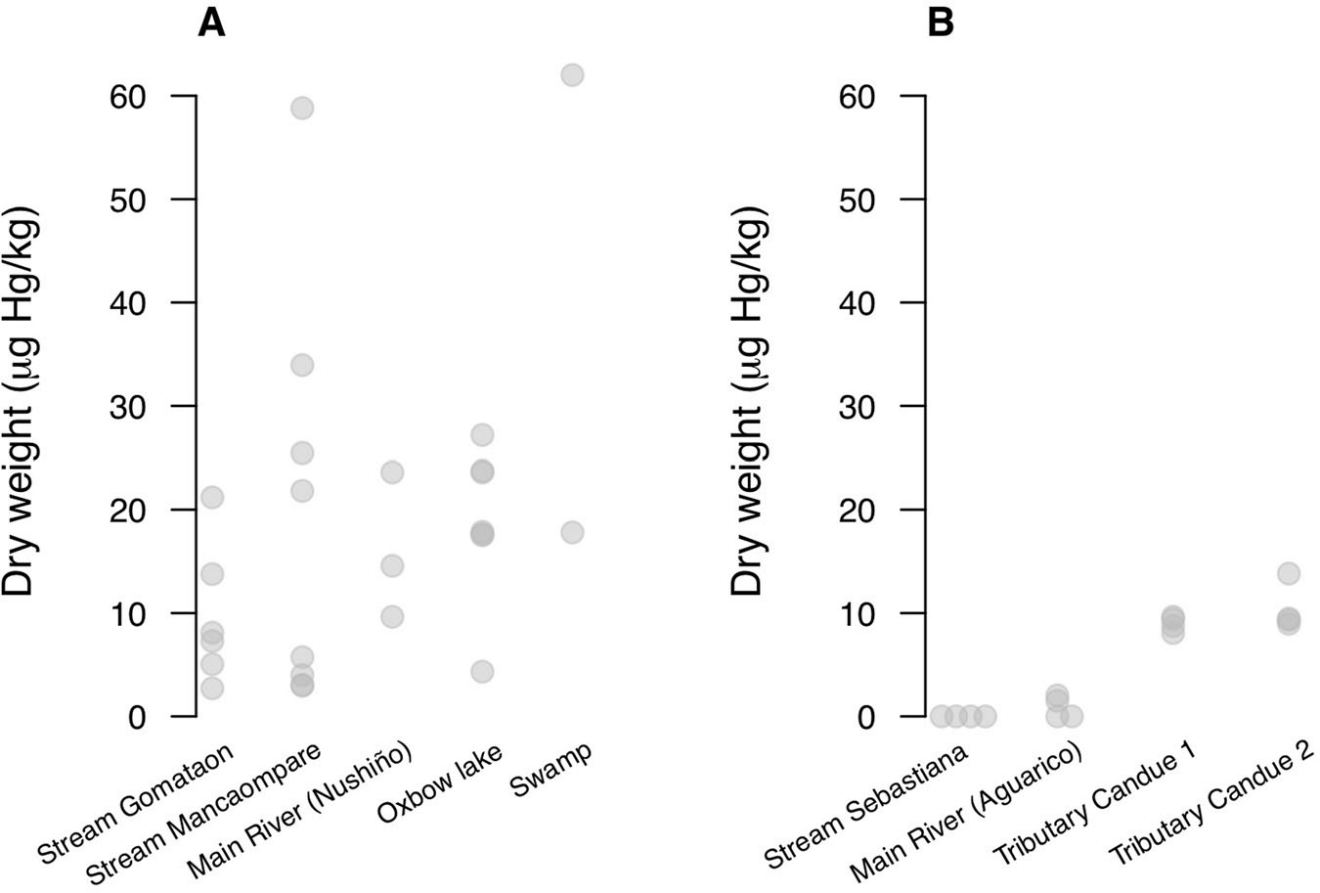
THg concentrations in sediments across different aquatic ecosystems from Indigenous communities’ territories of Ecuadorian Amazon: Gomataon (**A**) and Sinangoe (**B**)

A total of 42 sediment and 26 water samples were analyzed. Sediment samples exhibited concentrations between <0.5 and 62.01 Hg (µg/kg) (Table 3). Sediment samples in Gomataon showed no significant differences in THg concentrations among different types of aquatic habitats (chi-squared = 4.803, df = 4, p = 0.3081) (Table S6). In Sinangoé, the THg concentration between aquatic habitats differed significantly (chi squared = 12.488, df = 3, p = 0.005**). Sediments collected from the tributary river (Candué River 1 and 2) showed higher THg concentrations than other aquatic habitats (Fig. 5, Table S7). The robust linear model was significant (Intercept = 19.67, t = 6.46), where Sinangoé showed a negative effect with a coefficient of −10.227 (t = −2.981), suggesting lower THg concentration in sediments. Notably, ecosystem type also played a role, with river, stream, and swamp showing coefficients of −2.9031 (t = −0.631), −9.810 (t = −2.402), and 20.232 (t = 3.134), respectively. River samples did not show significant differences with sediments from oxbow lakes, but stream and swamp samples showed lower THg concentrations. Mercury (THg) concentrations in all sediment samples from aquatic ecosystems in this study were below the permissible limit of USA and Canada guidelines (486 µg/Kg) (CCME 1999) (Table 2).

**Table 3:** Results from THg and TOC analysis in sediments and THg in water of aquatic ecosystems from two indigenous communities in the Ecuadorian Amazon

Aquatic ecosystems	Locality	Sediments					Water				
			Hg		TOC			Hg		TOC	
		n	(µg/kg)	SD	(%)	SD	n	(µg/L)	SD	(mg/L)	SD
Gomataon											
Main river	Nushiño River up	2	19.09	6.39	0.50	0.19	2	<0.50	–	1.85	0.28
Main river	Nushiño River down	1	9.64	–	0.18	–	2	<0.50	–	–	–
Stream 1	Gomataon stream	7	13.15	0.33	0.42	0.27	2	1.72^a^	0.88	<0.60	–
Stream 2	Mancaompare stream	7	17.41	20.53^b^	0.77	0.46	2	<0.50	–	1.77	0.22
Oxbow lake	Oxbow lake	7	18.84	7.43	1	0.33	6	<0.50	–	2.05	0.48
Swamp	Swamp	2	39.91	31.26^c^	10.27	0.66	4	2.49	0.18	3.17	0.82
Sinangoé											
Main river	Aguarico river	4	1.77^d^	0.37	0.02	0.04^e^	2	2.49^f^	0.34	2.04^g^	–
Tributary river 1	Candué river 1	4	8.97	0.71	0.18	0.07	2	<0.5	–	2.81^g^	–
Tributary river 2	Candué river 2	4	10.39	2.3	0.3	0.14	2	<0.5	–	1.61^g^	–
Stream 1	Sebastiana stream	4	<0.5	–	0.001	0.001	2	<0.5	–	1.62^g^	–

Values show the number of samples analyzed (n), the average value measured, and the standard deviation (SD)

^a^The highest value is 1.72 µg/kg, and the lowest is <0.5 µg/L. Thus, the large standard deviations

^b^The highest Hg concentration measured is 58.81 µg/kg, and the lowest is 2.97 µg/kg

^c^The highest value is 62.01 µg/kg, and the lowest is 17.80 µg/kg. Thus, the large standard deviations

^d^4 samples were analyzed; 2 showed concentrations for which the mean and SD were shown, and 2 others were below the detection level, i.e., <0.5 µg/kg

^e^The highest value is 0.078%, and the lowest is 0.02%. Thus, the large standard deviations

^f^4 samples were analyzed; 2 showed concentrations for which the mean and SD were shown, and 2 other samples were below the detection level, i.e., <0.5 µg/L

^g^n = 1

THg concentration in 62% of water samples was below the detection limit of 0.50 µg/L and 7% below the permissible limit on water for human consumption according to Ecuadorian legislation (6 µg/L) and the WHO (2 µg/L). One water sample from the swamp in Gomataon and one from the main river (Aguarico River) in Sinangoé presented THg concentrations above 2 µg/L (Table 3).

### Mercury and organic carbon

**Fig. 6:**
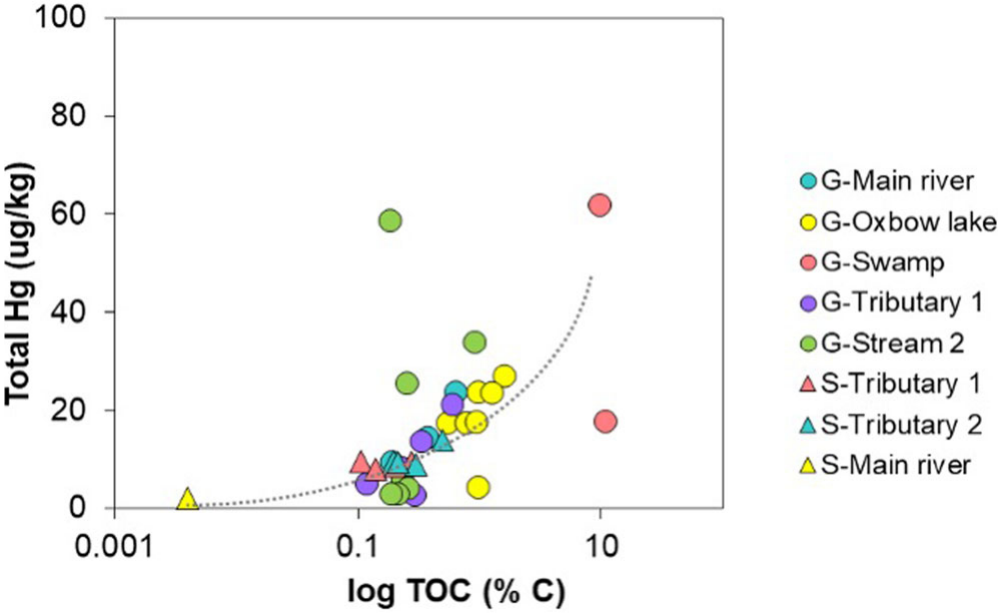
Total Organic Carbon (TOC %) and THg concentration from sediment samples of aquatic ecosystems in two Indigenous communities’ territories (G Gomataon, S Sinangoé) in Ecuadorian Amazon

Total organic carbon (%) in sediments was exponentially related to THg concentration from all aquatic ecosystems (R2 = 0.3421 m Y = 0.1323e^0.058x^) (Fig. 6). This relationship is driven mainly by the concentration of THg in sediments from the swamp, where there is high TOC, in Gomataon. The main river (Aguarico River) in Sinangoé showed the lowest concentrations of THg and TOC in sediments. Overall, sediments in Gomataon showed higher concentrations of THg and TOC than those in Sinangoé.

### Community surveys

Surveys showed that all interviewed community members of both areas catch and eat fish at least once a week (Table 4). Survey data confirmed that fishing in both communities is for subsistence. Members of both territories do not capture fish to market in other communities or in nearby cities.

**Table 4:** Survey results from closed questions in each community regarding their relationships and views on consumed fish species

	Gomataon		Sinangoé
Gender	M	5	7
	F	3	7
Activities	Fish merchant	0	27.78%
	Hunter/Fisherman	42.86%	11.11%
	Merchant	7.14%	5.56%
	Farmer	42.86%	16.67%
	Housewife	7.14%	22.22%
	Community guard	0	16.67%
Home members	Average	4.12	4.41
Consumption frequency	Daily	0	28.57%
	Weekly	100%	42.86%
	Every 15 days	0	28.57%
	Other	0	0
When do you fish	Morning	30%	26.67%
	Afternoon	70%	53.33%
	Evening	0	20.00%
Fishing frequency	Daily	0	0
	Weekly	100%	84.62%
	Every 15 days	0	7.69%
	Monthly	0	7.69%
	Other	0	0
Fishing method	Gill net	0	11.54%
	Hook	80%	50%
	Harpoon	20%	7.69%
	Hook lines	0	0
	Longline	0	0
	Castnet	0	30.77%
	Other	0	0

Survey results reveal that both communities consume several fish species. In Gomataon, at least 27 species are consumed, while in Sinangoé, only 16 species. In Gomataon, the most mentioned species in surveys were: *P. blochii* (omnivore), *Calophysus macropterus* (omnivore) and *Schizodon fasciatus* (herbivore) (Figs. S1, S3). In Sinangoé, the most mentioned species were *P. nigricans* (detritivore), *Chaetostoma* spp (detritivore), *Pseudoplatystoma fasciatum* (piscivore) and *S. hilarii* (piscivore). The Gomataon community consumes a greater diversity of fish species than Sinangoé (Fig. S2). Apart from fish, indigenous communities often rely on another source of protein. From aquatic ecosystems, they consume caimans in both areas, including *Melanosuchus niger*, *Caiman crocodilus*, and *Paleosuchus trigonatus*. To a lesser extent, they also eat turtles, turtle eggs, shrimp, and waterbirds (Fig. S5). Wild game meat from land includes mainly species of birds and mammals. Indigenous communities perceived the existence of several threats to fish (Fig. S6), including harmful fishing techniques, the use of dynamite, mullein (“barbasco”) and other poisonous chemicals. Other threats are directly related to the pollution from mining, water waste sewage, solid wastes, and gasoline and oil spills.

## Discussion

This study measured total mercury presence in aquatic ecosystems of two indigenous territories of the Ecuadorian Amazon. The obtained results provide new information from previously uncharacterized sites within the Napo River Basin. Our results suggest that the presence and absence of ASGM activities were not associated with mercury concentrations in fish, sediments, and water from the two different aquatic ecosystems, the Nushiño River (Gomataon community) and the Aguarico River (Sinangoé community), both tributaries of the Napo River.

### Sampled fish

All captured species occur in the Ecuadorian Amazon (Barriga 2012), where at least 36 species are known to be exploited for commercial fisheries (Burgos 2019). In addition, the most abundant families captured in our sampling (i.e., Characidae, Loricariidae, Pimelodidae) are also families with high species diversity in the Amazonian slope of Ecuador (Aguirre et al. 2021). Based on our observations during field trips and previous experiences in other localities, indigenous communities would eat any fish species regardless of size or flavor. Large-bodied species like *P. nigricans* or *C. macropterus* are preferred by indigenous communities (Jácome-Negrete 2013; Jácome-Negrete et al. 2018) while, smaller species like *A. unicolor*, *A. tetrametus*, and *P. blochii* are also part of subsistence fisheries (Sirén 2011; García et al. 2014; Jácome-Negrete et al. 2018). Unfortunately, we did not capture giant pimelodid catfishes in these sampling campaigns, except for a single individual of *Aguarunichthys torosus* in Sinangoé. Giant pimelodid catfishes are commonly targeted and exploited among indigenous communities (Jácome-Negrete 2012); however, longer and more targeted sampling campaigns are needed to capture these fish.

### Mercury in fish

High concentrations of mercury in piscivores and larger specimens were expected, given that bioaccumulation occurs in higher trophic guilds and with greater fish size (Beltran-Pedreros et al. 2011; Buck et al. 2019). For Gomataon, it is worth noting that all the specimens of the omnivore *C. macropterus* exhibited very high THg concentrations (669.21–2428.17 µg Hg/kg) and was the only species that surpassed the permissible limit for the Ecuadorian legislation (Fig. S3). This species has repeatedly been found to have high levels of THg throughout the Amazon (Beltran-Pedreros et al. 2011); its consumption has recently been banned in Brazil and Colombia (Bonilla-Castro 2022). Possibly, the high THg values of this species influenced the result from Gomataon, where piscivores don’t have an effect on the covariance analysis. Other species with high concentrations (Table S3, Fig. S3) in our study have been repeatedly reported with high mercury concentrations elsewhere in Ecuador: *B. cuvieri* (609–1340 µg Hg/kg) (De Matos et al. 2021; Souza-Araujo et al. 2022), *Salminus spp* (370–2847 µg Hg/kg) (Tuomola et al. 2008; Savassi et al. 2016), *Serrasalmus rhombeus* (614–1200 µg Hg/kg) (De Matos et al. 2021), and *Pimelodus* sp (1800 µg Hg/kg) (Hacon et al. 2020). THg concentrations in fish have also been reported (Silva et al. 2019), in fluvial systems with anthropogenic-prompted erosion activities (Lino et al. 2019; Barocas et al., 2023) and in lentic systems with high aquatic vegetation cover (Sampaio da Silva et al. 2009).

The intertwined hydro-geomorphological processes and anthropogenic activities may interact in combination to influence THg concentrations in aquatic ecosystems, and such interactions could explain the varying trophic guild concentrations found in fish from the studied sites. For instance, Ecuador’s highly vertical and horizontal dynamism of the Piedmont Amazon rivers reveals strong erosion patterns in the Aguarico (Sinangoé community) and Nushiño Rivers (Gomataon community). In the case of the Aguarico River, floods and flash events expose several soil horizons by increasing the increase of water levels and vertically mixing sand and pebbles. The Nushiño River, in contrast, shows less vertical dynamism, but a highly horizontal movement, exposing soil horizons in new margins created by flooding and meandering. Overall, our results agree with previous research that found high levels of mercury in consumed fish species in the Ecuadorian Amazon (Webb et al. 2004) (Table 5).

**Table 5:** Review of available research conducted in the Ecuadorian Amazon that reported the presence of THg in different samples from water, soil/sediment, fish, and human hair

Sample Type	THg values	Region	River Basins	Reference
Water	0.5–11.2 µg/L	NA	Misahualli, Pashimbi, Colonso and Pano	(Capparelli et al. 2020)
Water	1.2 µg/L	SA	Nangaritza	(González-Merizalde et al. 2016)
Water	<LOQ – 2.62 µg/L	MA	Nushiño	This study
Water	<LOQ – 3.18 µg/L	NA	Aguarico	This study
Soil	0–300 Hg µg/kg	NA	Napo River, Coca	(Mainville et al. 2006)
Soil	1700 Hg µg/kg	SA	Nambija	(Razmírez Requelme et al. 2003)
Sediment	2700 Hg µg/kg	SA	Nambija	(Razmírez Requelme et al. 2003)
Sediment	0–400 Hg µg/kg	NA	Misahualli, Pashimbi, Colonso and Pano	(Capparelli et al. 2020)
Sediment	20–1200 Hg µg/kg	SA	Nambija, Zamora, Yacuambi and Nangaritza	(Mora et al. 2019)
Sediment	0–500 Hg µg/kg	SA	Yacuambi	(López-Blanco et al. 2015)
Sediment	20–1100 Hg µg/kg	SA	Nangaritza To	(González-Merizalde et al. 2016)
Sediment	2.72–62.01 Hg µg/kg	MA	Nushiño	This study
Sediment	0–13.82 Hg µg/kg	NA	Aguarico	This study
Piscivorous fish	15–2970 Hg µg/kg	NA	Napo	(Webb et al. 2004)
Piscivorous fish	73–374 Hg µg/kg	NA	Napo	(Webb et al. 2015)
Piscivorous fish	54–841.09 Hg µg/kg	MA	Nushiño	This study
Piscivorous fish	68.66–926.72 Hg µg/kg	NA	Aguarico	This study
Non-piscivorous fish	4–290 Hg µg/kg	NA	Napo	(Webb et al. 2004)
Non-piscivorous fish	2.78–2428.17 Hg µg/kg	MA	Nushiño	This study
Non-piscivorous fish	7.06–112.19 Hg µg/kg	NA	Aguarico	This study

<LOQ = below the limit of quantification

Ecuadorian Amazonian Regions: *NA* Northern Amazon, *MA* Middle Amazon, *SA* Southern Amazon

### TOC and mercury

Research indicates a robust linear relationship between THg or MeHg and dissolved organic carbon (DOC) in freshwater environments (Lavoie et al. 2019). Higher organic carbon controls mercury distribution in freshwater bodies, and it’s known that carbon and mercury cycles are directly connected in the Amazon River basin (Belzile et al. 2008; Maia et al. 2009; Jardim et al. 2010), where there is a positive relationship indicated by the influence of geological and soil formation (Bonotto et al. 2018). Furthermore, mercury leaching from Amazonian soil reservoirs (e.g., due to deforestation) can be a significant pathway to mercury enrichment in rivers and other aquatic ecosystems (Fadini and Jardim 2001).

The correlation between THg concentration and TOC in sediments of aquatic ecosystems from Gomataon (Fig. 6) suggests that more significant carbon has a greater role in controlling mercury availability in the Piedmont Amazon of Ecuador. Hydro-geomorphological processes in Gomataon could also contribute to explain natural mercury availability compared to Sinangoé, as Gomataon resembles the ecosystems of the alluvial plains in the lower Amazon River Basin (i.e., oxbow lakes, swamps) (Noh et al. 2020; Lebel et al. 1998). Greater concentrations of TOC and THg in sediments would likely result in a more significant reduction of inorganic Hg^2+^ to organic MeHg by methanogenic aquatic bacteria, which could translate into the increased mercury concentrations in fish from Gomataon. Thus, surrounding ecosystems could influence carbon and mercury variation between the two studied areas. For example, aquatic vegetation covered in periphyton exhibited higher rates of Hg-methylation than near-bottom samples in the Tapajós River, likely because of more significant activity of reducing bacteria in these habitats (Davée Guimarâes et al. 2000). Aquatic vegetation, characteristic of floodplain ecosystems, was typical in swamps and oxbow lake Gomataon.

### Proximate causes for mercury differences

The contrasting THg concentrations in sediment between the two indigenous communities seem to be correlated with the presence of TOC, rather than with the presence/absence of ASGM. Notably, ASGM has not been reported in Gomataon and nearby communities, possibly due to the substrate of the Nushiño River, which consists of clay-sand and may not be ideal for such activities (Fig. 1). To better understand the proximate causes of mercury presence in Gomataon, future research should fill knowledge gaps regarding the occurrence of air deposition from oil exploitation, mining, and deforestation on a larger scale within the Curaray-Nushiño headwater basin (Fig. 1). In addition, to elucidate the differences in THg between the two indigenous community’s territories, we need to understand the natural occurrence of mercury along the altitudinal gradient Amazonian slope of Ecuador. The range of aquatic ecosystems should consider many aquatic ecosystems, as observed in Gomataon, where organic carbon might play a significant role in mercury availability (Table 3). To further understand mercury pathways and dynamics on a spatial-temporal scale, studies should also include an isotope analysis to confirm the source of Hg to develop a potential framework of THg mobilization through environmental compartments reaching aquatic ecosystems and humans.

Migratory and widely distributed fish like *S. hilarii* (Esguícero and Arcifa 2010), *C. macropterus* (Bonilla-Castillo et al. 2022), *P. blochii* (Kerguelén-Durango and Atencio-García 2015) and *A. torosus* (Stewart 1986) exhibited high THg. The incidence of migratory fish with high levels of mercury can be attributed to multiple causes, including pollution of aquatic ecosystems elsewhere from where fish migrate, feed, or mate (Reis et al. 2020). Non-migratory piscivore species like *Hoplias malabaricus* found in Gomataon did not exhibit high mercury concentrations, which agrees with previous research on this species in Ecuador and Perú, showing focalized high levels of mercury only in areas where oil spills have occurred (Webb et al. 2015). Gomataon and their surroundings have not experienced any oil spills in the past.

Finally, considering the fluctuation of mercury concentrations in aquatic ecosystems and its components throughout hydrological cycles (flood-pulso) (de Castro-Paiva et al. 2022), future studies should conduct multiple samplings throughout the year to comprehensively assess temporal variations in mercury levels. We acknowledge that conducting one sampling campaign limits our ability to gain a thorough understanding of the dynamics of mercury cycling and bioaccumulation in these regions.

### Surveys

Indigenous communities’ weekly consumption of fish agrees with other studies in the Ecuadorian Amazon (Webb et al. 2004), where fish are the primary source of protein, followed by wild-game protein (i.e., mammals and birds) (Sirén and Machoa 2008). In Sarayaku territory (Kichwa indigenous nationality), lying north of Gomataon, fish constitute two-thirds of the total dietary fat and almost half of the dietary protein (Sirén 2004). Wild fish consumption is also positively related to how isolated communities are from urban areas (Vasco and Sirén 2019), consistent with our results in Gomataon, where fish was consumed every week. In contrast, people from Sinangoé mentioned fishing or eating fish only once every 15 days (Table 2). Sinangoé relies on other protein sources, ranging from wild game to, goods from nearby cities, and from domestic animals. To build on this result, people in Sinangoe mentioned occasional access to other proteins due to its proximity to urban areas. The survey results also revealed that most fish species mentioned by communities (i.e., Pimelodids and Loricariids) are known to be solely for subsistence in many indigenous communities of Ecuador and have been reported in communities from the Curaray River (Jácome-Negrete 2013).

Similarly, *P. nigricans*, *Pseudoplatystoma* spp., and *Brachyplatistoma* spp., are widely known migratory species with high value for indigenous communities’ diet, throughout the Napo River Basin (Sirén 2004; Neira et al. 2006; Jácome-Negrete 2012; García et al. 2014). Overall, both communities preferred species that were common and that were easier- to- catch species than others, such as the omnivores *P. blocchii* and *C. macropterus* in Gomataon and *P. nigricans* and *S. hilarii* in Sinangoé. Regarding the difference in the diversity of consumed fish species between communities, this could be attributed to the location of each community. Sinangoé is located at an altitude of 500 m a.s.l., while Gomataon is at 200 m a.s.l. This naturally limits fish species distributions that end in the foothills of the Andes around 300 m a.s.l. (Ibarra and Stewart 1989; Galacatos et al. 1996).

## Conclusions

In this study, we analyzed the presence of mercury in fish, sediments, and water samples in two indigenous communities in the Piedmont Amazon of Ecuador. We found that, when examining mercury levels in fish tissues across diverse habitats, species, trophic guilds, and sizes, no significant differences were detected between the two communities, challenging the notion that mining presence/absence plays a central role in determining the risk of mercury exposure in this study. Predictors of greater THg concentrations, such as trophic guild and size, agreed with our expectations at a local level. Significantly, indigenous communities frequently consume all species in this study. Five percent of samples showed higher levels above recommended values by the WHO, but most of fish exhibited THg below the permissible limits. Three out of five species (four piscivores and one omnivore), with THg above the WHO limit, were found in Gomataon, where communities depend more on fish for subsistence. The effects of fish consumption in these communities are unknown yet, and sampling of THg on local people is needed to assess the potential impacts. For sediments, all samples had THg concentrations below the recommended values for aquatic life. Sediments in Gomataon presented higher THg concentrations than Sinangoé, suggesting an important role of TOC from aquatic ecosystems such as swamps and oxbow lakes present in Gomataon, where the community does also fish. The majority of THg concentrations in water samples from both communities presented lower concentrations than the USEPA permissible limits (2 µg/L), except for the swamp at Gomataon and the main river (Aguarico River) at Sinangoé that showed average concentrations above the international permissible limit for drinking water.

The implications of our results are essential for the well-being of the communities, especially considering the importance of aquatic ecosystems and particularly fish consumption in riverine indigenous communities. Results from Gomataon are particularly relevant for actions since their diet depends heavily on fish. Our results suggest that future studies should focus on understanding mercury’s impact on local human populations across the Ecuadorian Amazon. Based on this study, future studies aiming at monitoring mercury should account for other triggers of potential sources of presence, as well as include other elements (i.e., iron, lead, arsenic, antimony) together with environmental variables (e.g., DOC) to better understand mercury dynamics across aquatic ecosystems and along the Andean-Amazonian altitudinal gradient. Future studies in the region should include isotope analysis to understand better mercury’s origins and fate in sediments and non-migratory fish and, as well as control sites to identify background mercury levels in the Ecuadorian Amazon.

### Supplementary information

Supplementary Information
