# Supplementary material for: Mercury in aquatic ecosystems of two indigenous communities in the Piedmont Ecuadorian Amazon: evidence from fish, water, and sediments
Source: Ecotoxicology. 2024 Jun 7;33(4-5):440–56. doi: 10.1007/s10646-024-02764-w (PMC11213792; doi:10.1007/s10646-024-02764-w)
Supplement: Supplementary file 1 — Supplementary Information [file 10646_2024_2764_MOESM1_ESM.docx]

**Supplementary materials**


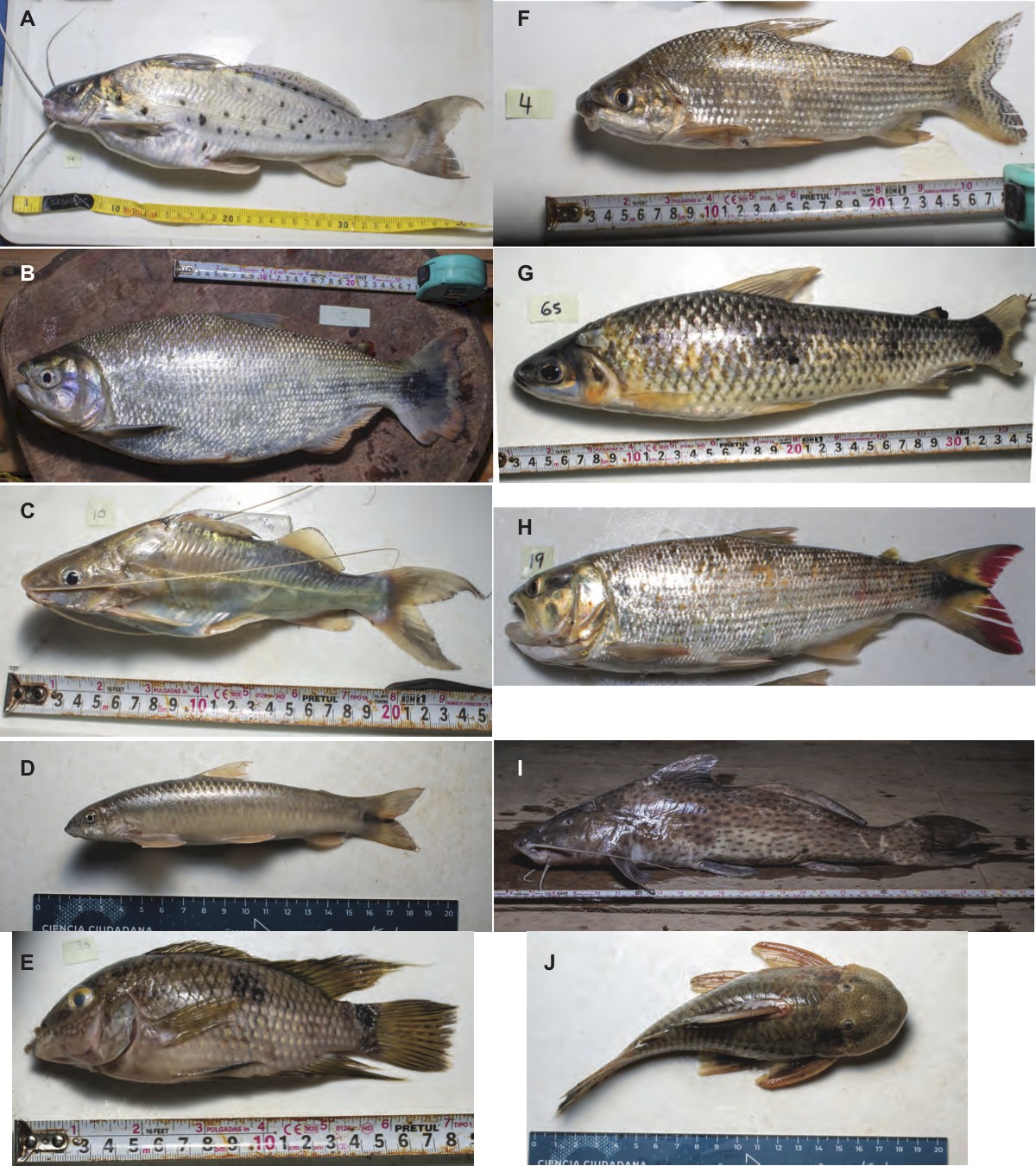


**Figure S1.** Representative fish species sampled in Gomataon and Sinangoé. A) *Calophysus macropterus*^1^, B) *Brycon hilarii*^1^ C) *Pimelodus blochii*^1^, D) *Parodon buckleyi*^2^, E. *Aequidens tetramerus*^1^, F) *Prochilodus nigricans*^1,2^, G) *Leporinus* sp^1^, H) *Salminus hilarii*^1,2^, I) *Aguarunichthys torosus*^2^, J) *Chaetostoma breve*^2^.

^1^ : Gomataon, ^2^ : Sinangoé

**A**

**
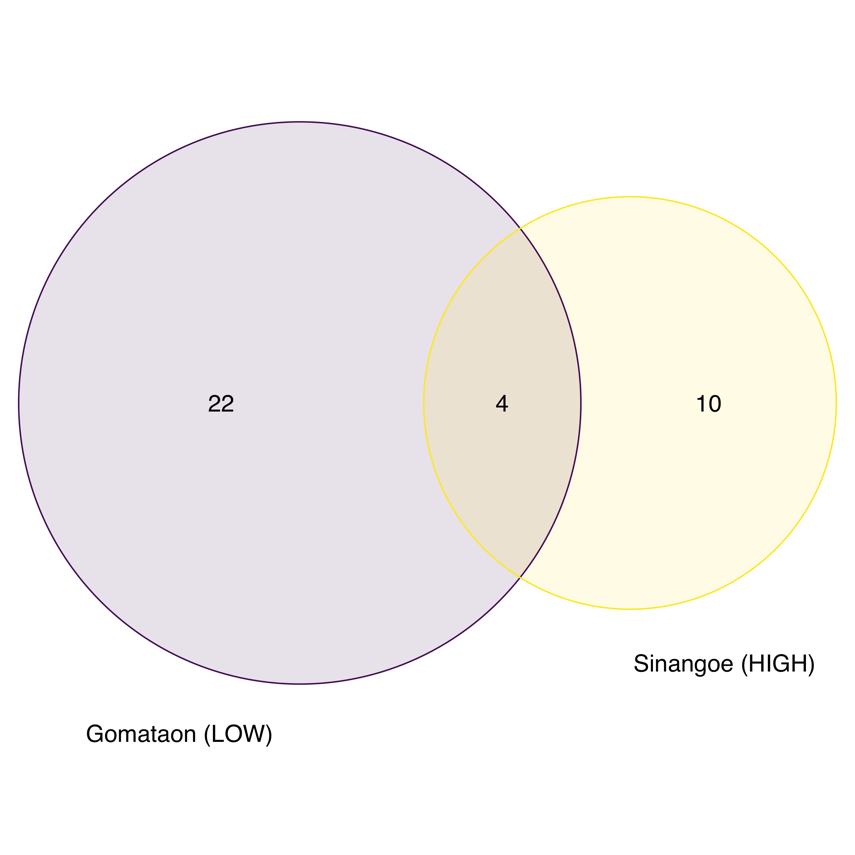
**

**B**

**Figure S2.** A) Freshwater fish families of sampled fish species. B) Venn Diagram of species sampled in the two studied areas.

**Figure S3**. Omnivorous and piscivorous species having moderate to high concentrations of THg from A) Gomataon and B) Sinangoé. Each circle represents and individual. Dashed gray lines represent recommended thresholds by different organizations.

**Figure S4.** Results from open-questions regarding which fish species are typically captured and consumed in both communities.

**Figure S5.** Results from the open-question regarding which other proteins, aside from fish, communities obtain from aquatic and terrestrial ecosystems.

**Figure S6.** Results from the open-question regarding which are the perceived threats to fish in each community.

**Table S1.** Sampled fish species in Gomataon

| Order | Family | Species | Nº individuals | Guild | Reference |
| --- | --- | --- | --- | --- | --- |
| Characiformes | Erythrinidae | *Hoplias malabaricus* | *3* | Piscivore | (Villares Junior and Goitein 2015) |
|  | Serrasalmidae | *Serrasalmus rhombeus* | *4* | Piscivore | (Froese and Pauly 2023) |
|  | Anostomidae | *Leporinus friderici* | *2* | Omnivore | (Froese and Pauly 2023) |
|  |  | *Leporinus fasciatus* | *1* | Omnivore | (Froese and Pauly 2023) |
|  |  | *Leporinus sp.* | *2* | Omnivore | (Froese and Pauly 2023) |
|  |  | *Schizodon fasciatus* | *2* | Herbivore | (Mortillaro et al. 2015) |
|  | Curimatidae | *Curimata aspera* | *1* | Detritivore | (Vari 1983) |
|  | Prochilodontidae | *Prochilodus nigricans* | *1* | Detritivore | (Froese and Pauly 2023) |
|  | Ctenolucidae | *Boulengerella cuvieri* | *4* | Piscivore | (Froese and Pauly 2023) |
|  | Triportheidae | *Triportheus angulatus* | *2* | Herbivore | (Marín-Erausquín Giulio and Aron-Neumann Alejandro 2006) |
|  | Bryconidae | *Brycon aff. melanopterus* | *6* | Herbivore | (Froese and Pauly 2023) |
|  |  | *Brycon hilarii* | *1* | Herbivores | (Reys et al. 2009) |
|  |  | *Salminus aff.hilarii* | *1* | Piscivore | (Froese and Pauly 2023) |
|  | Characidae | *Astyanax aff. bimaculatus* | *5* | Omnivore | (Froese and Pauly 2023) |
|  |  | *Creagrutus aff. amoenus* | *3* | Insectivore | (Bojsen, B.H. y Barriga 2002) |
| Siluriformes | Loricariidae | *Hypostomus hemicochliodon* | *6* | Detritivore | (van der Sleen and Albert 2018) |
|  |  | *Panaqolus aff. nocturnus* | *4* | Detritivore | (van der Sleen and Albert 2018) |
|  |  | *Aphanotorulus unicolor* | *14* | Detritivore | (van der Sleen and Albert 2018) |
|  | Heptateridae | *Pimelodella sp* | *3* | Omnivore | (van der Sleen and Albert 2018) |
|  | Heptateridae | *Rhamdia sp.* | *1* | Omnivore | (van der Sleen and Albert 2018) |
|  | Pimelodidade | *Calophysus macropterus* | *4* | Omnivore | (GARCÍA VÁSQUEZ et al. 2017) |
|  |  | *Megalonema* sp. | *1* | Omnivore | Based on *M. platycephalym* |
|  |  | *Pimelodus albofasciatus* | *2* | Omnivore | Based on *P. blochii* |
|  |  | *Pimelodus blochii* | *26* | Omnivore | (Froese and Pauly 2023) |
| Cichliformes | Cichlidae | *Aequidens tetramerus* | *10* | Insectivore | (da Costa and Soares 2015) |
|  |  | *Chrenicichla sp.* | *3* | Piscivore | (van der Sleen and Albert 2018) |
| Myliobatiformes | Potamotrygonidae | *Potamotrygon sp.* | *1* | Insectivore | Based on *P. motoro* (Almeida et al. 2010) |

**Table S2.** Sampled fish species in Sinangoé

| Order | Family | Species | Nº individuals | Guild | Reference |
| --- | --- | --- | --- | --- | --- |
| Characiformes | Parodontidae | *Parodon buckleyi* | *7* | Detritivore | (Zamudio et al. 2008) |
|  | Hemiodontidae | *Hemiodus unimaculatus* | *2* | Detritivore | (Froese and Pauly 2023) |
|  | Curimatidae | *Steindachnerina guentheri* | *1* | Detritivore | (van der Sleen and Albert 2018) |
|  | *Prochilodontidae* | *Prochilodus nigricans* | *18* | Detritivore | (Froese and Pauly 2023) |
|  | Characidae | *Salminus aff.hilarii* | *16* | Piscivore | (Froese and Pauly 2023) |
|  |  | *Astyanax bimaculatus* | *2* | Omnivore | (Froese and Pauly 2023) |
|  |  | *Astyanax* sp. | *20* | Omnivore | Based on A. bimaculatus |
|  |  | *Charax* aff. *tectifer* | *1* | Piscivore | (van der Sleen and Albert 2018) |
|  |  | *Hemibrycon* aff. *polyodon* | *6* | Omnivore | (van der Sleen and Albert 2018) |
|  |  | *Cynopotamus amazonum* | *1* | Piscivore | (van der Sleen and Albert 2018) |
| Siluriformes | *Loricariidae* | *Chaetostoma breve* | *12* | Detritivore | (van der Sleen and Albert 2018) |
|  | *Pimelodidade* | *Aguarunichthys torosus* | *1* | Piscivore | Local knowledge |
|  |  | *Pimelodus albofasciatus* | *1* | Omnivore | Based on *P. blochii* |
| Cichliformes | *Cichlidae* | *Oreochromis niloticus* | *1* | Herbivore | (Froese and Pauly 2023) |

*****For simplicity, in the detritivore category we also included fish that feed on algae

**Table S3.** Trophic guild per aquatic ecosystem.

| Ecosystem | Trophic Guild | | | | |
| --- | --- | --- | --- | --- | --- |
|  | Piscivore | Omnivore | Herbivore | Detritivore | Insectivore |
| Gomataon |  |  |  |  |  |
| Main River | X | X | X | X |  |
| Oxbow Lake |  | X | X | X |  |
| Stream | X | X | X |  | X |
| Swamp | X |  |  |  |  |
| Sinangoe |  |  |  |  |  |
| River 4th order | X | X |  | X |  |
| Main River | X | X | X | X |  |
| Stream | X | X |  | X |  |

**Table S4.** Concentrations of THg in analyzed fish species from the Waorani community of Gomataon, Nushiño River (Curaray Basin).

| No. | | Species | n | | Avg. | SD | Range | | | Avg. | SD |
| --- | --- | --- | --- | --- | --- | --- | --- | --- | --- | --- | --- |
|  |  | | |  | Dry weight | Technical replica | Wet weight | | | Wet weight | Sample replica |
|  |  | | |  | (µg Hg/kg) |  | (µg Hg/kg) | | | (µg Hg/kg) |  |
| 1 | *Calophysus macropterus* | | | 4 | 7274.0 | 0.01 | 2428.2 | – | 669.2 | 1490.1 | 635.4 |
| 2 | *Salminus aff.* | | | 1 | 2696.3 | 0.01 |  |  |  | 841.1 |  |
| 3 | *Boulengerella cuvieri* | | | 4 | 1898.8 | 0.01 | 583.7 | – | 217.5 | 487.2 | 155.8 |
| 4 | *Megalonema sp.* | | | 1 | 1744.0 | 0.00 |  |  |  | 360.2 |  |
| 5 | *Brycon amazonicus* | | | 1 | 1007.9 | 0.01 |  |  |  | 258.8 |  |
| 6 | *Rhamdia sp.* | | | 1 | 1247.7 | 0.00 |  |  |  | 241.6 |  |
| 7 | *Serrasalmus rhombeus* | | | 4 | 768.2 | 0.02 | 320.1 | – | 83.0 | 188.2 | 98.8 |
| 8 | *Pimelodus blochii* | | | 26 | 810.4 | 0.01 | 332.3 | – | 36.6 | 158.8 | 63.3 |
| 9 | *Schizodon fasciatus* | | | 2 | 646.8 | 0.01 | 235.6 | – | 69.1 | 152.4 |  |
| 10 | *Leporinus friderici* | | | 2 | 671.3 | 0.01 | 273.4 | – | 26.7 | 150.1 | 123.3 |
| 11 | *Pimelodus albofasciatus* | | | 2 | 716.7 | 0.01 | 174.6 | – | 117.7 | 146.1 |  |
| 12 | *Brycon aff. melanopterus* | | | 6 | 416.3 | 0.01 | 180.7 | – | 100.1 | 125.0 | 30.8 |
| 13 | *Potamotrygon sp.* | | | 1 | 649.2 | 0.04 |  |  |  | 92.3 |  |
| 14 | *Hoplias malabaricus* | | | 3 | 373.9 | 0.01 | 109.1 | – | 54.0 | 80.8 | 22.5 |
| 15 | *Curimata aspera* | | | 1 | 321.5 | 0.01 |  |  |  | 75.1 |  |
| 16 | *Aequidens tetramerus* | | | 10 | 261.0 | 0.01 | 95.9 | – | 54.1 | 71.1 | 13.4 |
| 17 | *Prochilodus nigricans* | | | 1 | 273.1 | 0.01 |  |  |  | 64.4 |  |
| 18 | *Triportheus angulatus* | | | 2 | 294.2 | 0.01 | 65.3 | – | 57.6 | 61.5 |  |
| 19 | *Chrenicichla sp.* | | | 3 | 290.8 | 0.02 | 81.1 | – | 37.5 | 58.6 | 17.8 |
| 20 | *Leporinus fasciatus* | | | 1 | 267.2 | 0.00 |  |  |  | 58.5 |  |
| 21 | *Leporinus sp.* | | | 2 | 183.6 | 0.01 | 47.7 | – | 33.8 | 40.8 |  |
| 22 | *Aphanotorulus unicolor* | | | 14 | 139.6 | 0.01 | 45.8 | – | 23.1 | 32.2 | 6.9 |
| 23 | *Astyanax aff. bimaculatus* | | | 5 | 106.8 | 0.01 | 38.2 | – | 14.0 | 24.0 | 10.8 |
| 24 | *Pimelodella sp.* | | | 3 | 110.2 | 0.02 | 23.1 | – | 15.2 | 18.0 | 3.6 |
| 25 | *Panaqolus aff. nocturnus* | | | 4 | 75.1 | 0.03 | 34.3 | – | 2.8 | 17.5 | 11.6 |
| 26 | *Creagrutus aff. amoenus* | | | 3 | 28.8 | 0.03 | 12.7 | – | 4.9 | 8.0 | 3.4 |
| 27 | *Hypostomus hemicochliodon* | | | 6 | 24.1 | 0.02 | 7.1 | – | 3.7 | 4.9 | 1.1 |

**Table S5.** Concentrations of THg in analyzed fish species from the Cofán community of Sinangoé, Aguarico River Basin.

| No. | Specie | n | Avg. | SD | Range | | | Avg. | SD |
| --- | --- | --- | --- | --- | --- | --- | --- | --- | --- |
|  |  |  | Dry weight | Technical replica | Wet weight | | | Wet weight | Sample replica |
|  |  |  | (µg Hg/kg) |  | (µg Hg/kg) | | | (µg Hg/kg) |  |
| 1 | *Aguarunichthys torosus* | 1 | 2997.4 | 12.1 |  |  |  | 548.2 |  |
| 2 | *Salminus hilarii* | 16 | 1587.8 | 17.1 | 926.7 | – | 151.61 | 336.3 | 203.2 |
| 3 | *Cynopotamus amazonum* | 1 | 1028.4 | 5.3 |  |  |  | 196.1 |  |
| 4 | *Hemiodus unimaculatus* | 2 | 554.3 | 7.0 | 96.73 | – | 85.61 | 79.2 |  |
| 5 | *Hemibrycon aff. polyodon* | 6 | 312.6 | 3.3 | 99.23 | – | 36.7 | 75.9 | 20.7 |
| 6 | *Charax aff. tectifer* | 1 | 285.3 | 4.7 |  |  |  | 68.7 |  |
| 7 | *Pimelodus albofasciatus* | 1 | 435.6 | 3.3 |  |  |  | 66.1 |  |
| 8 | *Prochilodus nigricans* | 18 | 304.0 | 4.5 | 105.88 | – | 40.20 | 62.9 | 20.5 |
| 9 | *Steindachnerina guentheri* | 1 | 407.1 | 29.3 |  |  |  | 56.5 |  |
| 10 | *Parodon buckleyi* | 7 | 193.3 | 3.5 | 112.19 | – | 24.-3 | 51.8 | 30.6 |
| 11 | *Astyanax sp.* | 20 | 190.1 | 2.7 | 73.05 | – | 12.1 | 39.9 | 17.0 |
| 12 | *Astyanax bimaculatus* | 2 | 257.2 | 2.9 | 50.07 | – | 25.74 | 37.9 |  |
| 13 | *Chaetostoma breve* | 12 | 55.4 | 1.9 | 44.09 | – | 7.06 | 15.9 | 9.1 |
| 14 | *Oreochromis niloticus* | 1 | 55.0 | 3.0 |  |  |  | 8.5 |  |

**Table S6.** Sediment samples and range of THg concentrations from Gomataon

| Sampling site | N | Range [Hg] ug/kg | $\overline{\text{X}}$ [Hg] | SD |
| --- | --- | --- | --- | --- |
| Swamp | 2 | 17.8 – 62.01 | 39.91 | 31.26 |
| Mancaompare stream | 7 | 2.97 – 58.81 | 17.41 | 20.53 |
| Oxbow lake | 7 | 4.33 – 27.19 | 18.84 | 7.43 |
| Nushiño River | 3 | 9.64 – 23.60 | 19.08 | 6.39 |
| Gomataon stream | 7 | 2.72 – 21.19 | 13.15 | 11.04 |

**Table S7.** Sediment samples and range of THg concentrations from Sinangoé

| Sampling site | N | Range [Hg] ug/kg | $\overline{\text{X}}$ [Hg] | SD |
| --- | --- | --- | --- | --- |
| Candue river 2 | 4 | 8.96 – 13.82 | 10.39 | 2.3 |
| Candue river 1 | 4 | 8.07 – 9.64 | 8.97 | 0.71 |
| Aguarico River | 4 | <LC – 2.03 | 1.77 | 0.37 |
| Sebastiana stream | 4 | <LD - <LD | NA | NA |

**File S1**

**Fisheries-research survey for Amazonian indigenous communities (USFQ/BRI/TNC)**

Name: ___________________________ Age: ______ Survey taker: ________________

Community: ________________________

Gender: Male Female Economic activities: Merchant fishermen

Hunter

Merchant

Farmer

Other _________________

Level of studies **____________________**

1. **Do you fish? Yes** Go through all the survey

Ç√

**No** Continue until #**6** and then start over at **#16**

1. **How many people are part of your household? ______**

Ç√

Ç√

1. **Do you eat fish? Yes No**
2. **How often?**

Daily Weekly Every 2 weeks Monthly Other **_____________**

1. **Which species did you eat/cook in the last 24h?**

| **Species** | **Type** (abundant seasonal or rare) | **Average size (cm)** | **Number of fish** | **Weight (kg)** | **Sale price (if applies)** |
| --- | --- | --- | --- | --- | --- |
|  |  |  |  |  |  |
|  |  |  |  |  |  |
|  |  |  |  |  |  |
|  |  |  |  |  |  |

If seasonal, specify which season (wet or dry).

1. **Which species do you usually consume?**

| **Species** | **Type** (abundant seasonal or rare) | **Average size (cm)** | **Number of fish** | **Weight (kg)** | **Sale price (if applies)** |
| --- | --- | --- | --- | --- | --- |
|  |  |  |  |  |  |
|  |  |  |  |  |  |
|  |  |  |  |  |  |
|  |  |  |  |  |  |
|  |  |  |  |  |  |
|  |  |  |  |  |  |
|  |  |  |  |  |  |
|  |  |  |  |  |  |
|  |  |  |  |  |  |
|  |  |  |  |  |  |
|  |  |  |  |  |  |

If seasonal, specify which season (wet or dry).

**Fishing activities**

1. **When do you fish? 8. How often?**

Morning Afternoon Night Daily Weekly Monthly

Every 2 weeks Other ___________

**9. Fish technique employed 10. Why do you fish?**

Nets / Gill nets Hooks Feeding Income

Harpoon Longline Enterntainment Other________

Castnet Other _____________

**11. Where do you fish? (Use the map)** ___________________________

**12.**  **Where do you sell the fish?** (if applies) ___________________________

**13. At what time do you fish?** ___________________________

**14. I would like to ask you about the fish you caught in the previous day. Which species did you fish in the last 24 hrs?**

Captured / sold species

| **Species** | **Type (**abundant seasonal or rare**)** | **Average size (cm)** | **Number of fish** | **Weight (kg)** | **Sale price (if applies)** | **Selling**  **(easy or hard)** |
| --- | --- | --- | --- | --- | --- | --- |
|  |  |  |  |  |  |  |
|  |  |  |  |  |  |  |
|  |  |  |  |  |  |  |
|  |  |  |  |  |  |  |
|  |  |  |  |  |  |  |

If seasonal, specify which season (wet or dry).

**15. Now I want to ask you more questions about all types of fish that you catch throughout the year. What species do you usually fish?**

| **Species** | **Type (**abundant seasonal or rare) | **Average size (cm)** | **Number of fish** | **Weight (kg)** | **Sale price (if applies)** | **Selling**  **(easy or hard)** |
| --- | --- | --- | --- | --- | --- | --- |
|  |  |  |  |  |  |  |
|  |  |  |  |  |  |  |
|  |  |  |  |  |  |  |
|  |  |  |  |  |  |  |
|  |  |  |  |  |  |  |

If seasonal, specify which season (wet or dry).

**16. Which are the most commonly consumed fish species?**

| **Species** | **Type** (abundant seasonal or rare) | **Average size (cm)** | **Number of fish** | **Weight (kg)** | **Sale price (if applies)** |
| --- | --- | --- | --- | --- | --- |
|  |  |  |  |  |  |
|  |  |  |  |  |  |
|  |  |  |  |  |  |
|  |  |  |  |  |  |
|  |  |  |  |  |  |

If seasonal, specify which season (wet or dry).

**17. Do you buy fish at a market/port? If yes, which ones?**

| **Species** | **Type** (abundant seasonal or rare) | **Average size (cm)** | **Number of fish** | **Weight (kg)** | **Sale price (if applies)** |
| --- | --- | --- | --- | --- | --- |
|  |  |  |  |  |  |
|  |  |  |  |  |  |
|  |  |  |  |  |  |
|  |  |  |  |  |  |
|  |  |  |  |  |  |

If seasonal, specify which season (wet or dry).

**18. Do you consume fish from aquaculture? If yes, which ones?**

**19. What do you think are the main threats for fish?**

**20. Are there mining activities or oil exploitation nearby? (use map if necessary)**

**21. What other proteins do you obtain from waterbodies? (e.g. turtles, eggs, caimans, aquatic mammals)**

**22. What are the main animal species that you eat from the forest? (add hunting season if possible)**

**Literature References:**

Bojsen, B.H. y Barriga R (2002) Effects of deforestation on fish community structure in Ecuadorian Amazon streams. Freshw Biol 47:2246–2260

da Costa ID, Soares MO (2015) The seasonal diet of aequidens tetramerus (Cichlidae) in a small forest stream in the machado river basin, rondônia, Brazil. Acta Amazon 45:365–372. https://doi.org/10.1590/1809-4392201500223

Froese R, Pauly D (2023) FishBase. In: World Wide Web electronic publication (www.fishbase.org). www.fishbase.org, version

GARCÍA VÁSQUEZ A, RUIZ GÓMEZ L, VARGAS DÁVILA G, et al (2017) ALIMENTACIÓN NATURAL DE LA MOTA Calophysus macropterus (Lichtenstein, 1819), EN AMBIENTES DE LA AMAZONÍA PERUANA. Folia Amazónica 26:29–36. https://doi.org/10.24841/fa.v26i1.416

Marín-Erausquín Giulio, Aron-Neumann Alejandro (2006) VARIACIÓN TEMPORAL DE LA DIETA DE «SARDINA» Triportheus angulatus (Characiformes, Characidae) EN UNA LAGUNA DE INUNDACIÓN DE LA SELVA AMAZÓNICA PERUANA. Folia Aamzónica 15:141–150

Mortillaro JM, Pouilly M, Wach M, et al (2015) Trophic opportunism of central Amazon floodplain fish. Freshw Biol 60:1659–1670. https://doi.org/10.1111/fwb.12598

Reys P, Sabino J, Galetti M (2009) Frugivory by the fish Brycon hilarii (Characidae) in western Brazil. Acta Oecologica 35:136–141. https://doi.org/10.1016/j.actao.2008.09.007

van der Sleen P, Albert JS (2018) Field guide to the fishes of the Amazon, Orinoco & Guianas. Princeton University Press, New Jersey

Vari RP (1983) Phylogenetic Relationships of the Families Curimatidae, and Chilodontidae (Pisces : Characiformes). Smithson Contrib Zool 378:

Villares Junior GA, Goitein R (2015) Variations of Salminus hilarii diet (Ostariophysi, Characidae): Seasonal and ontogenetic effects. Brazilian Journal of Biology 75:574–580. https://doi.org/10.1590/1519-6984.17213

Zamudio J, Urbano-Bonilla A, Maldonado-Ocampo JA, et al (2008) Hábitos alimentarios de diez especies de peces del piedemonte del departamento del Casanare, Colombia. Rev Asoc Colomb Ictiol 10:43–55
